# Thermal treatment of magnetite nanoparticles

**DOI:** 10.3762/bjnano.6.143

**Published:** 2015-06-23

**Authors:** Beata Kalska-Szostko, Urszula Wykowska, Dariusz Satula, Per Nordblad

**Affiliations:** 1 Institute of Chemistry, Hurtowa 1, 15-399 Białystok, Poland; 2 Faculty of Physics, ul. K.Ciołkowskiego 1L, 15-245 Białystok, Poland; 3 Uppsala University, Ångströmlaboratoriet, Lägerhyddsv. 1, Box 534, 751 21 Uppsala, Sweden

**Keywords:** high temperature corrosion, internal oxidation, IR spectroscopy, metal matrix composites, Mössbauer spectroscopy, X-ray diffraction

## Abstract

This paper presents the results of a thermal treatment process for magnetite nanoparticles in the temperature range of 50–500 °C. The tested magnetite nanoparticles were synthesized using three different methods that resulted in nanoparticles with different surface characteristics and crystallinity, which in turn, was reflected in their thermal durability. The particles were obtained by coprecipitation from Fe chlorides and decomposition of an Fe(acac)_3_ complex with and without a core–shell structure. Three types of ferrite nanoparticles were produced and their thermal stability properties were compared. In this study, two sets of unmodified magnetite nanoparticles were used where crystallinity was as determinant of the series. For the third type of particles, a Ag shell was added. By comparing the coated and uncoated particles, the influence of the metallic layer on the thermal stability of the nanoparticles was tested. Before and after heat treatment, the nanoparticles were examined using transmission electron microscopy, IR spectroscopy, differential scanning calorimetry, X-ray diffraction and Mössbauer spectroscopy. Based on the obtained results, it was observed that the fabrication methods determine, to some extent, the sensitivity of the nanoparticles to external factors.

## Introduction

Nanostructured magnetite has become one of the most investigated materials due to its unusual magnetic properties. In addition, it is recognized as an inert compound that is almost entirely nontoxic to living organisms [1]. Apart from that, iron and its oxides on the nanometer scale can possess superparamagnetic properties, allowing for their application in various fields. The list of possible applications encompasses biomedical engineering, MRI contrast agents, hyperthermia treatment, sensing and biosensing [2–3]. They are also very promising candidates for electrical-related applications, for example, energy and magnetic storage materials, sensors and catalysts [4], or even environmental remediation or sieves [5]. For this reason, the study of the physical properties and the chemical and thermal stability of ferrite nanoparticles is of crucial importance [6]. Moreover, magnetite nanostructures can be relatively easy to obtain by a simple synthetic procedure [7]. All of the above-mentioned advantages promote the popularity of ferrite nanoparticles. Additionally, there is the possibility to fabricate many different forms of iron oxides. During each step of the synthesis process, a structure transformation can be expected, resulting in various magnetic properties, and therefore, application potential. It has been observed that even at the synthesis level, due to the oxidation process, a structural transformation can be expected. This brings about new features of the magnetic properties which can be applied [8]. In addition, surface modification by the deposition of a chemically dissimilar layer (e.g., Ag) can be of particular importance, especially in the case of bio-related applications [9]. Another critical problem faced by researchers is that it is very difficult to obtain nanoparticles with exactly the same well-defined size, morphology and shape from different synthetic methods. As far as the applications are concerned, the production of a desired size and good characterization of the obtained magnetic nanoparticles are very important factors. To obtain a desired range of diameters, strict control of the reaction conditions, such as the synthesis time, temperature, concentration of the reactants and added surfactants, must be maintained. Furthermore, it has been observed that the size of the particles increases with extended synthesis time [8]. On the other hand, the particle size can be modified by using surfactants with various carbon chain lengths [10–12].

Few types of nanoparticles have been extensively studied over large temperature ranges. It has been shown that in some systems, at room temperature, the nanoparticles tend to exhibit spontaneous growth [13]. This phenomenon is called anomalous grain growth. On the other hand, it has been shown that for particular granular powders, the thermal stability increases with larger grain size [14]. These size-related effects are explained by enthalpy and stress, which influences the activation energy value [15]. Thermal sensitivity depends, in most cases, mainly on the type of nanomaterial and method of fabrication [16].

As for all nanomaterials, the properties of magnetite change on the nanoscale. At the bulk level, the oxidation of magnetite to hematite at room temperature is inhibited, and only by heating to 600 °C can changes in the crystalline structure be achieved [17]. At the nanoscale level, changes in the crystalline structure can be expected and observed at much lower temperatures, even very close to the room temperature. This is related to surface enthalpy and activation energy, which are size dependent [18]. It was found that Fe nanoparticles oxidize to a mixture of iron oxides (γ-Fe_2_O_3_ and α-Fe_2_O_3_) even at 200 °C [19]. However, this temperature can vary due to the high surface area and various activity of the nanoparticles, which results in a more exothermic heat process during oxidation at low temperature. In general, it can be assumed that phase transformation in nano-granular systems occurs from 200 to 600 °C with different contribution from both oxides, γ-Fe_2_O_3_ and α-Fe_2_O_3_ [20]. It should also be underlined that the data regarding the behavior of nanosystems at elevated temperatures are very different and generalizations cannot be made [16].

In this paper, tests of three different kinds of magnetite nanoparticles and their behavior at higher temperatures starting from 50 °C up to 500 °C have been selected. Some changes in differential scanning calorimetry (DSC) cycles have been observed in our previous measurements [21] for short temperature cycles. However, these changes were not very specific. To observe possible particle phase transformations, heat treatment was performed for 24 h.

## Experimental

### Materials and apparatus

To obtain the various magnetite nanoparticles, the following chemicals were purchased from Aldrich and Fluka: Fe(acac)_3_, 1,2-hexadecanediol, phenyl ether, FeCl_3_·6H_2_O, FeCl_2_·4H_2_O, AgNO_3_, NH_3_ solution and oleic acid; tetrabutylammonium hydroxide (TBAOH), 1-octadecanol and oleylamine, respectively. The cleaning and rough size separation of the nanoparticles were performed by the magnetic field separation method with the use of acetone, sonic bath treatment and a permanent magnet. The thermal treatment was performed in an air flow furnace.

FTIR spectra were collected in reflection mode at room temperature (RT) using a Nicolet 6700 Infrared spectrometer working in the spectral range between 500–4000 cm^−1^. The quality of the nanopowders was observed using a Tecnai G2 X-TWIN transmission electron microscope (TEM). The analysis of the crystal structure was carried out using an Agilent Technologies SuperNova X-ray diffractometer (XRD) with a Mo microfocused source (Mo Kα = 0.713067 Å). The thermal analysis of the magnetite and core–shell nanoparticles was performed on a Mettler Toledo differential scanning calorimeter (DSC). A Quantum Design MPMS SQUID magnetometer was used for the magnetization measurements. Mössbauer spectra (MS) were obtained using a conventional spectrometer working in constant acceleration mode at RT with a CoCr radioactive source.

### Preparation of magnetite nanoparticles

As was previously mentioned, the tested magnetite nanoparticles (MNP-1, MNP-2, MNP-3) were obtained by three different synthetic methods. The first one is based on coprecipitation of iron(II) and iron(III) chlorides in aqueous ammonia solution (MNP-1). This is a low temperature reaction that was conducted at 80 °C under Ar atmosphere. The procedure was adopted from the Massart method [7,22–23].

The second (MNP-2) and third (MNP-3) type of nanoparticles were based on the layer-by-layer synthetic procedure, where thermal decomposition of iron(III) acetylacetone salts in a phenyl ether solution was provided. This reaction was prepared using the following conditions: −230 °C, oxygen-free environment, and Ar flow. The main points of the described synthesis were first proposed by Sun et al. and further details can be found there [24–25].

The nanoparticles obtained from the Fe(acac)_3_ complex were also modified with a silver shell (third synthetic procedure, MNP-3). In this case, magnetite nanoparticles in the reaction solution were mixed with the following reactants: AgNO_3_ (2 mmol)_,_ 1,2-hexadecanediol (1 mmol), phenyl ether (20 mL) and oleylamine (2.5 mmol). The solution was mixed for 15 min at RT, then for 2 h at 30 °C, and at the end for 30 min at 140 °C [26–27]. When the solution was cooled down, the nanoparticles were separated from the solution with a permanent magnet, washed in deoxygenated acetone and dried to powder form with a vacuum evaporator.

## Results and Discussion

### Gravimetric results

**Figure 1:**
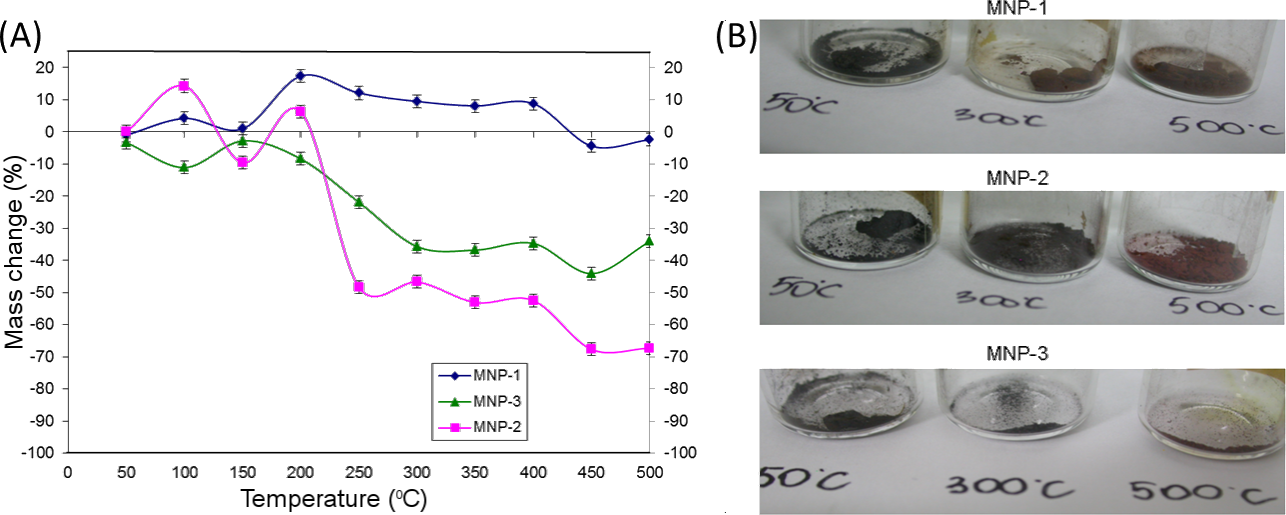
(A) Mass change of the nanoparticle powders (%) with respect to the temperature variation. (B) Color change of the nanoparticles after heating.

The resulting precipitates were dried to powder form in a vacuum evaporator. The obtained nanoparticles were thermally treated in an oven in the temperature range 50–500 °C for 24 h with a temperature step of 50 °C. The as-prepared nanoparticles were pretested using the techniques presented in this paper. Starting from 50 °C, before and after heating, the powder mass was checked and the weight change is plotted in Figure 1A. A gradual color change of the powder upon heating was simultaneously observed, which is depicted in Figure 1B.

For all of the nanoparticles, a gradual transition from black to reddish brown was observed (see Figure 1B). However, this did not occur at the same temperature for all samples. A much more rapid color transformation was noticed in the case of the MNP-2 particles in comparison to MNP-1. The coating of the magnetite nanoparticles with a silver shell (MNP-3) shows that the color change is less pronounced and not as intense as in the case of the uncoated particles. This implies that an additional metallic layer changes the thermal stability, and therefore, partial protection from the oxidation process is achieved.

This observation was also confirmed by the mass change, where it can be seen that MNP-3 particles show less pronounced changes in mass in comparison to MNP-2 and the mass loss is shifted toward higher temperatures. The MNP-1 sample had almost no mass change over the studied temperature range. In Table 1, the details of the mass changes of the nanoparticles before and after heating are collected.

**Table 1:** Mass change of nanoparticles before and after heat treatment.

Temperature [°C]	MNP-1			MNP-2			MNP-3		
	Before [g] ±0.005	After [g] ±0.005	Mass difference [%]^a^ ±0.5	Before [g] ±0.005	After [g] ±0.005	Mass difference [%]^a^ ±0.5	Before [g] ±0.005	After [g] ±0.005	Mass difference [%]^a^ ±0.5

50	0.098	0.097	−1.0	0.091	0.091	0.0	0.095	0.092	−3.3
100	0.095	0.099	+4.2	0.084	0.096	+14.3	0.097	0.087	−11.1
150	0.101	0.102	+1.0	0.094	0.085	−9.6	0.098	0.095	−2.8
200	0.092	0.108	+17.4	0.095	0.101	+6.3	0.099	0.091	−8.4
250	0.091	0.102	+12.1	0.091	0.067	−48.4	0.098	0.077	−21.9
300	0.095	0.104	+9.5	0.090	0.048	−46.7	0.099	0.063	−35.7
350	0.099	0.107	+8.1	0.096	0.045	−53.1	0.099	0.063	−36.8
400	0.091	0.099	+8.8	0.099	0.035	−52.5	0.099	0.065	−34.7
450	0.098	0.094	−4.4	0.099	0.032	−67.7	0.100	0.065	−44.1
500	0.099	0.097	−2.4	0.101	0.033	−67.3	0.098	0.065	−34.1

^a^+ mass increase; − mass decrease.

First of all, it can be seen that the MNP-1 nanoparticle system is much more stable over the studied temperature range. An observed difference in mass is smaller than 20%. Over the temperature range 50–425 °C, a slight increase in the particle weight is observed. This could be due to the adsorption of oxygen on the particle surface, which penetrates to the core of the nanoparticle. At higher temperatures the release of these gases from the surface is possible. For this series of particles, only a slight modification of the powder color is observed. For the MNP-2 particles, a more rapid change in color was observed, accompanied by a drastic decrease of the powder weight in the temperature range 200–300 °C. The difference in temperature stability for both types of particle cores can be connected with a difference in the morphology of individual particles. Precipitation from chlorides (MNP-1) leads to the formation of a well-defined monocrystalline structure [28], while thermal decomposition of Fe(acac)_3_ (MNP-2 and MNP-3) causes the growth of rather polycrystalline particles. This was especially clearly observed when larger particles were grown and can be expected from the synthesis. The existence of interfaces between separate crystallites inside each particle can be the reason for a faster oxidation process. Such a scenario is in good agreement with the speculation that polycrystallinity causes the presence of grain boundaries, which significantly influences the stability/susceptibility to the oxide. On the other hand, in the case of polycrystalline particles, their structure is also less dense and oxygen can penetrate more easily inside the particle along the edges of structural discontinuation. Also, the surface chemistry is different from the previous case and it can behave differently at elevated temperature where partial evaporation could occur. The comparison of MNP-3 with MNP-2 particles shows that the mass loss is much smaller, only up to 44% (450 °C for MNP-2), in comparison to 67% for MNP-3. For MNP-3 nanoparticles, we have not observed any mass increase during heating. This also proves that metallic shell strengthens the thermal stability of the nanoparticles by sealing the surface, which prevents oxygen penetration.

### TEM microscopy

**Figure 2:**
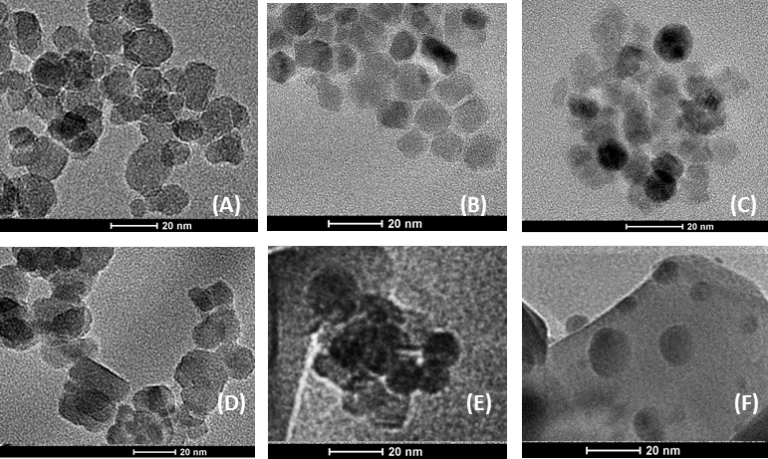
TEM images of magnetite nanoparticles before (as-prepared) (A, B, C) and after the heating process at 500 °C ( D, E, F), for MNP-1, MNP-2, and MNP-3, respectively.

The morphology of magnetite nanopowders before (as prepared) and after heating at 500 °C was observed by TEM on the powdered sample supported on a 400 mesh Cu grid covered by amorphous carbon. The obtained images are depicted in series in Figure 2.

In Figure 2, six images of nanoparticles are depicted. Figure 2A shows MNP-1 before heating and Figure 2D after heat treatment at 500 °C for 24 h. The comparison of these images leads to the conclusion that there is not much difference in shape between the particles before and after heating. The crystalline structure, the shape and the size of the nanoparticles has been preserved. Figure 2B shows the as-prepared MNP-2 nanoparticles in Figure 2E, where these nanoparticles are presented after heating under the same conditions as in the previous case. The comparison of these two images allows for the observation that the morphology of the nanoparticles also does not change after heat treatment. Only the lateral distribution is much worse. For the MNP-3 series, the appearance of the particles after heating is the most different from the as-prepared case but the size and shape of the structures are maintained.

### X-ray diffraction

**Figure 3:**
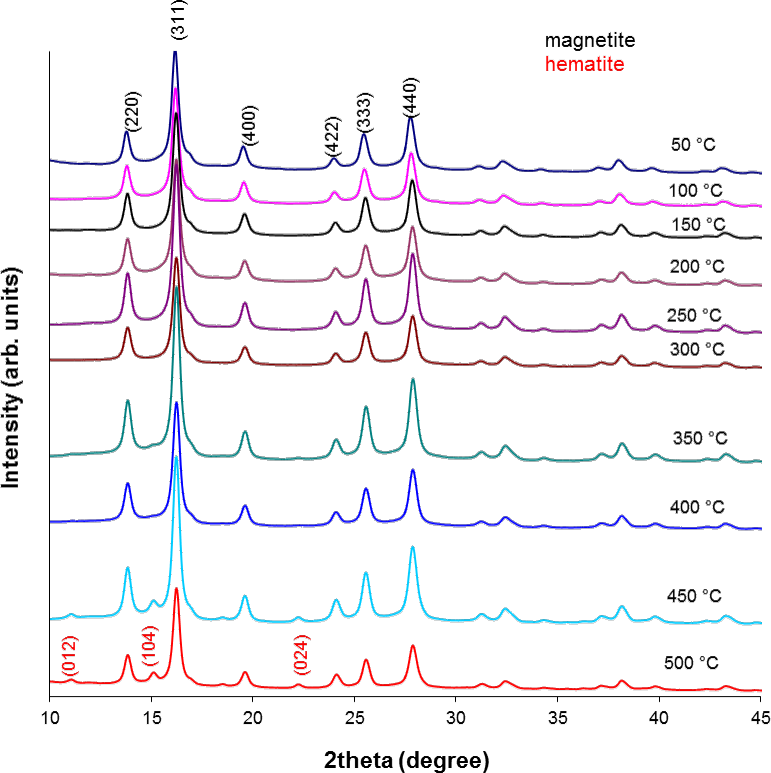
X-ray pattern of MNP-1 nanoparticles after the heating process.

**Figure 4:**
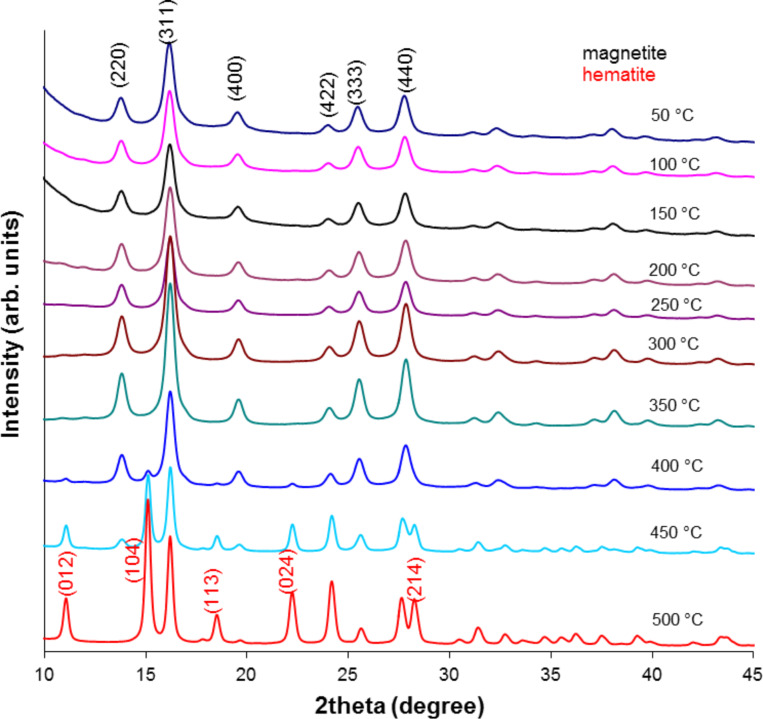
X-ray pattern of MNP-2 nanoparticles after the heating process.

**Figure 5:**
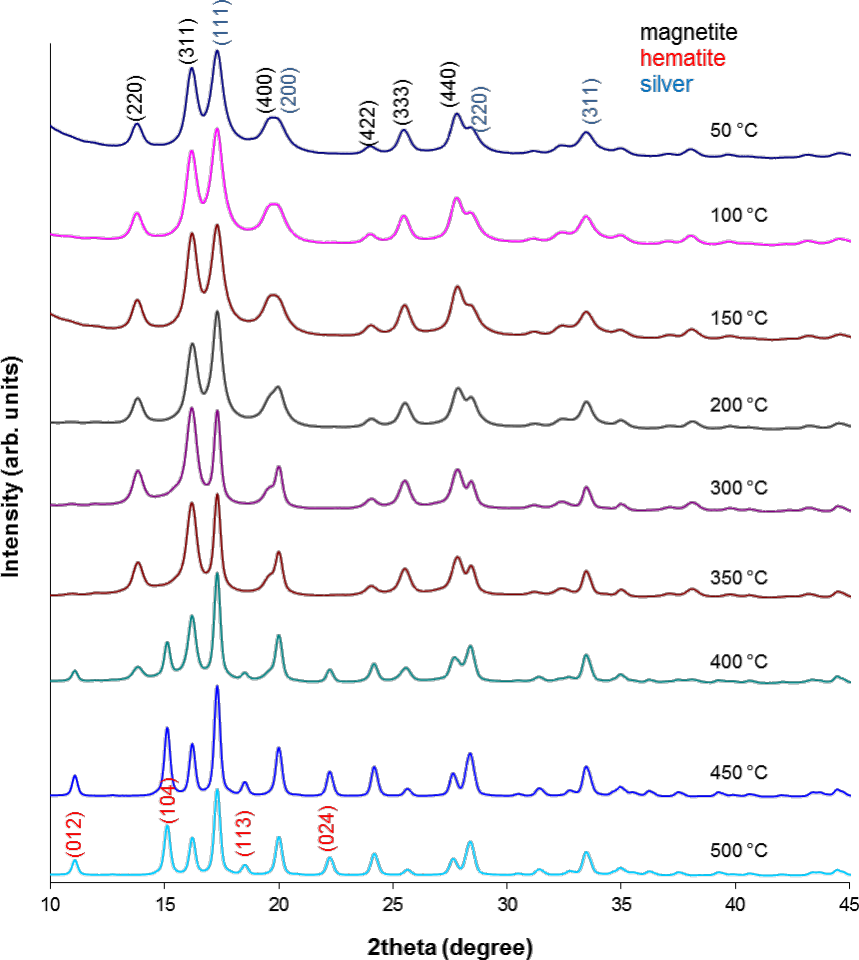
X-ray pattern of MNP-3 nanoparticles after the heating process.

All thermally tested nanoparticles were measured by XRD to observe the evolution in their crystalline structure in the studied temperature range. The obtained results in the series are depicted in Figures 3–5.

Here, in each set of XRD patterns, the well-defined crystalline structure of magnetite and/or maghemite with indexes (*hkl*) ascribed to (220), (311), (400), (422), (333) and (440) [12,29] are observable. MNP-1 nanoparticles do not show any significant amount of additional structure up to 400 °C and therefore no additional patterns are presented. At 450 °C and 500 °C a small amount of hematite develops. Nevertheless, the intensity of the respective peaks is relatively low. However, MNP-2 nanoparticles develop strong signals even at 400 °C, which are typical for the hematite structure and are indexed as (012), (104), (113), (024) and (214) [30]. Their intensity is as high as that observed at 500 °C for the previous series. The MNP-3 particles are also stable only up to 400 °C, and above this temperature the hematite structure can be seen together with the magnetite/maghemite phase and metallic Ag (111), (200), (220) and (311) [31]. This proves that the MNP-2 nanoparticles are less stable with respect to temperature, especially at highest tested temperatures, in comparison to MNP-1. A lower sensitivity to external factors for the MNP-2 particles was also observed with regards to aqueous solution stability [20].

To determine the average grain size of the nanoparticles before and after heating, a quantitative analysis was performed using Scherrer’s equation [32]:

[1]
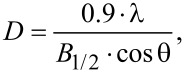


where *D* is the grain size (Å), λ is the wavelength (Mo source, 0.7136 Å), *B*_1/2_ is the full width at half maximum intensity of the peak (rad), and θ is the diffraction angle (rad). The calculated results for these particles are compiled in Table 2.

**Table 2:** Estimated grain sizes of thermally treated nanoparticles, determined by X-ray diffraction.

Temperature	MNP-1 grain size [nm] ±2	MNP-2 grain size [nm] ±2	MNP-3 grain size [nm] ±2

As-prepared	16	11	11
50 °C	18	12	10
100 °C	17	13	11
150 °C	18	13	11
200 °C	19	12	10
250 °C	20	12	11
300 °C	16	13	13
350 °C	18	13	12
400 °C	17	13	14
450 °C	17	18	18
500 °C	15	24	25

The results presented in Table 2 show that MNP-1 particles preserve their average particle size regardless of temperature treatment, while MNP-2 and MNP-3 particles tend to increase the average diffracting zone above 400 °C (which can be suggested by the aggregation of the particles). This observation again proves that the two proposed synthetic procedures result in differing quality of the particle core. The discrepancy between the TEM average particle size and that calculated from Equation 1 originates from the fact that different factors (e.g., nonlinear detector parameters, structural defects, tension, composition variation, crystal imperfections, etc.) significantly contribute to the broadening of the line width of the XRD patterns [33]. Therefore, the average particle size calculated this way is only a rough estimation. The temperature dependence of the line width also shows that heat tends to relax the imperfection of the particle core, and therefore, the average particle size appears much larger than it actually is [34]. The temperature treatment also relaxes some imperfection of the crystals, which at lower temperature cause line broadening.

### Differential scanning calorimetry

**Figure 6:**
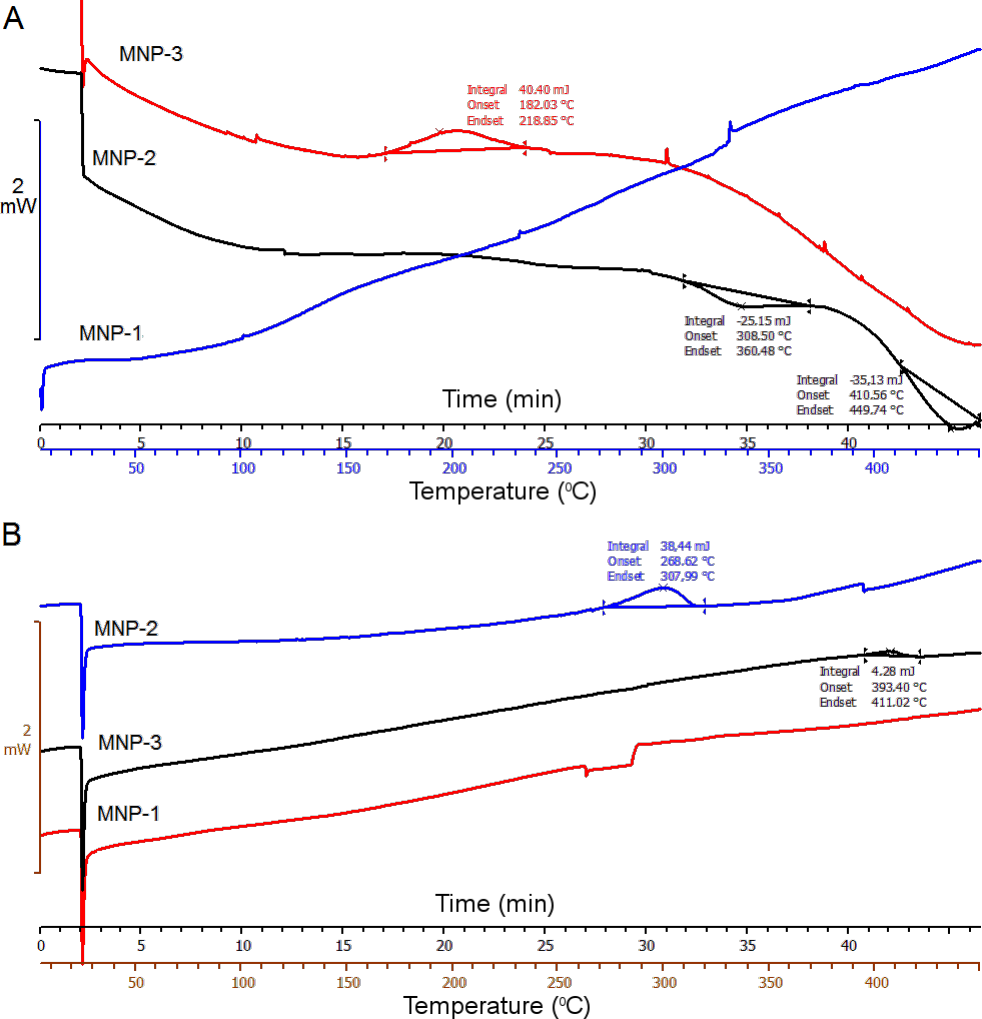
DSC curves of reference nanoparticles before heating (A) and after heating at 500 °C (B).

A quick (1 hour) heating and cooling cycle in the temperature range 2–450 °C with a scan rate of 10 °C/min was conducted for extreme samples. As a reference, an empty pan was heated at the same time. For each DSC measurement, a quantity of about 2 mg of the nanoparticle powder was sealed in an Al crucible. The collected temperature scan curves are depicted in Figure 6.

Figure 6 presents the DSC curves of the three studied types of nanoparticles. In Figure 6A we can see reference thermal cycles of nanoparticles before the heating process. In Figure 6B nanoparticles heated up to 500 °C for 24 h in each case are collected. As can be seen, the reference curves show few thermal changes. MNP-3 nanoparticles show one exothermic process over the temperature range 182–218 °C with 40.4 MJ of energy. For the MNP-2 nanoparticles, two endothermic processes appear above 300 °C: the first at 306–360 °C and the second at 410–449 °C, with −25 and −35 MJ of energy, respectively. MNP-1 nanoparticles do not present any thermal changes, which implies that they are not modified in the studied temperature range and that they have better thermal stability in comparison to the other particles. The temperature cycles of nanoparticles after the heating process are a bit different compared to the unheated ones. At first, MNP-3 shows only very small exothermic process at 393–411 °C, while MNP-2 exhibits an exothermic process at 266–307 °C with 38 MJ of energy (in contrast to the reference curve). The MNP-1 curve also does not show any thermal changes. The described results are in good agreement with XRD studies, which showed that thermal changes and oxidation of MNP-2 and MNP-3 particles start much faster than in case of MNP-1. The DSC data also confirm the gravimetric results where for MNP-1 nanoparticles the weight change with temperature remains almost constant, while MNP-2 and MNP-3 exhibit changes. Therefore, the DSC curve slopes before heat treatment are different for MNP-1, MNP-2 and MNP-3. The observed behavior suggests the opposite curve slope trend between those samples, which is in agreement with the gravimetric results. The comparison of the DSC curves between the samples after heating for 24 h at 500 °C suggests that the surface and/or core modification no longer takes place – the slope of each line is the same. This result explains the gravimetric results and supports the scenario that for MNP-1, surface ligands are rigidly fixed to the sample and do not contain any weakly adsorbed solvents. However, MNP-2 and MNP-3 most likely contain residuals of solvents that gradually evaporate, which is not observed for the samples measured after heat treatment.

### IR spectroscopy

**Figure 7:**
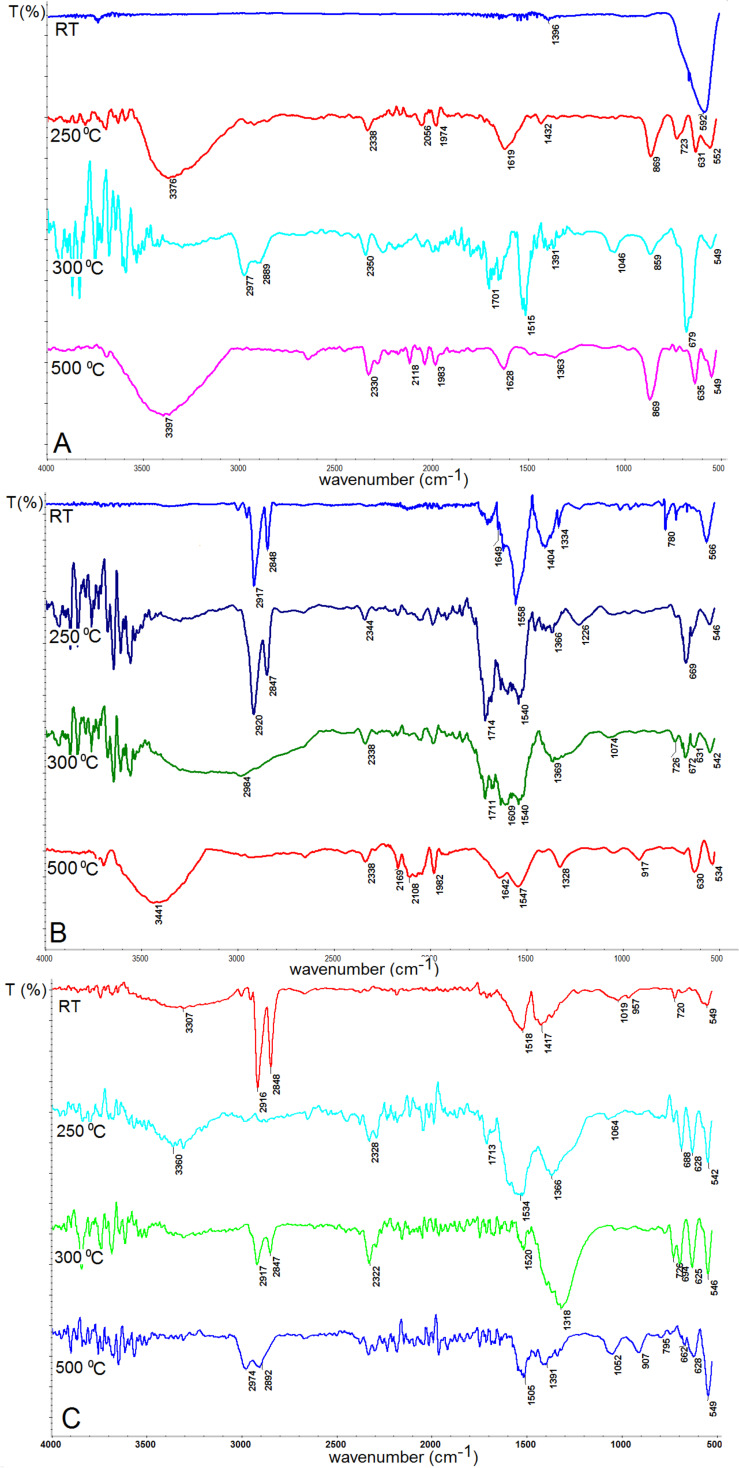
IR spectra of magnetite nanoparticles (A) MNP-1, (B) MNP-2, (C) MNP-3 before and during the heating process.

Magnetite nanoparticles were further tested by IR spectroscopy to observe changes taking place on the nanoparticles surface due to temperature treatment. The representative spectra are collected in Figure 7.

Figure 7 shows the selected IR spectra of the nanoparticles after heat treatment in an oven in the temperature range from 50 to 500 °C. In Figure 7A we observe the IR spectra of MNP-1 nanoparticles, in Figure 7B for the MNP-2 sample, and in Figure 7C for MNP-3. Heating resulted in oxidation of magnetite firstly to maghemite then to hematite for all types of nanoparticles. When the particles are heated to temperatures up to 150 °C, Fe–O bonds typical for Fe_3_O_4_ are still preserved (560–580 cm^−1^) [6]. Nevertheless, in almost every spectrum, oxidation to hematite (540 cm^−1^), maghemite (647–679 cm^−1^) [35], lepidocrocite (730 and 1060 cm^−1^) [36], goethite (860 cm^−1^) [37] and ferrihydrite (964 cm^−1^) [35] is well observed, which suggests a slow surface oxidation process. The other bands reported in Figure 7A belong to N–H bonds originating from TBAOH used during the synthesis procedure (1515 cm^−1^ and 2164–2654 cm^−1^). For the as-prepared sample, there are C=O bonds present typical for acetone (1710 cm^−1^) and those present in the carbon chain in the TBAOH solution (2900–2974 cm^−1^) [38]. In Figure 7B we can also observe bands connected with the presence of Ar–O–Ar bonds (1233 cm^−1^), acetylacetonate groups (1455–1558 cm^−1^), –COOH bonds (1705 cm^−1^) [38] and carbon chain bonds (2850–2920 cm^−1^) [39] from oleic acid. The increasing intensity of the –OH band (3200–3600 cm^−1^) with temperature is observed in both types of nanoparticles. It can be explained as a relation to oxidation process. After the 500 °C heat treatment, a shift in the 556–534 cm^−1^ band toward lower wavenumbers is observed, which is in good agreement with expectations and hematite formation [36].

### SQUID magnetometry

**Figure 8:**
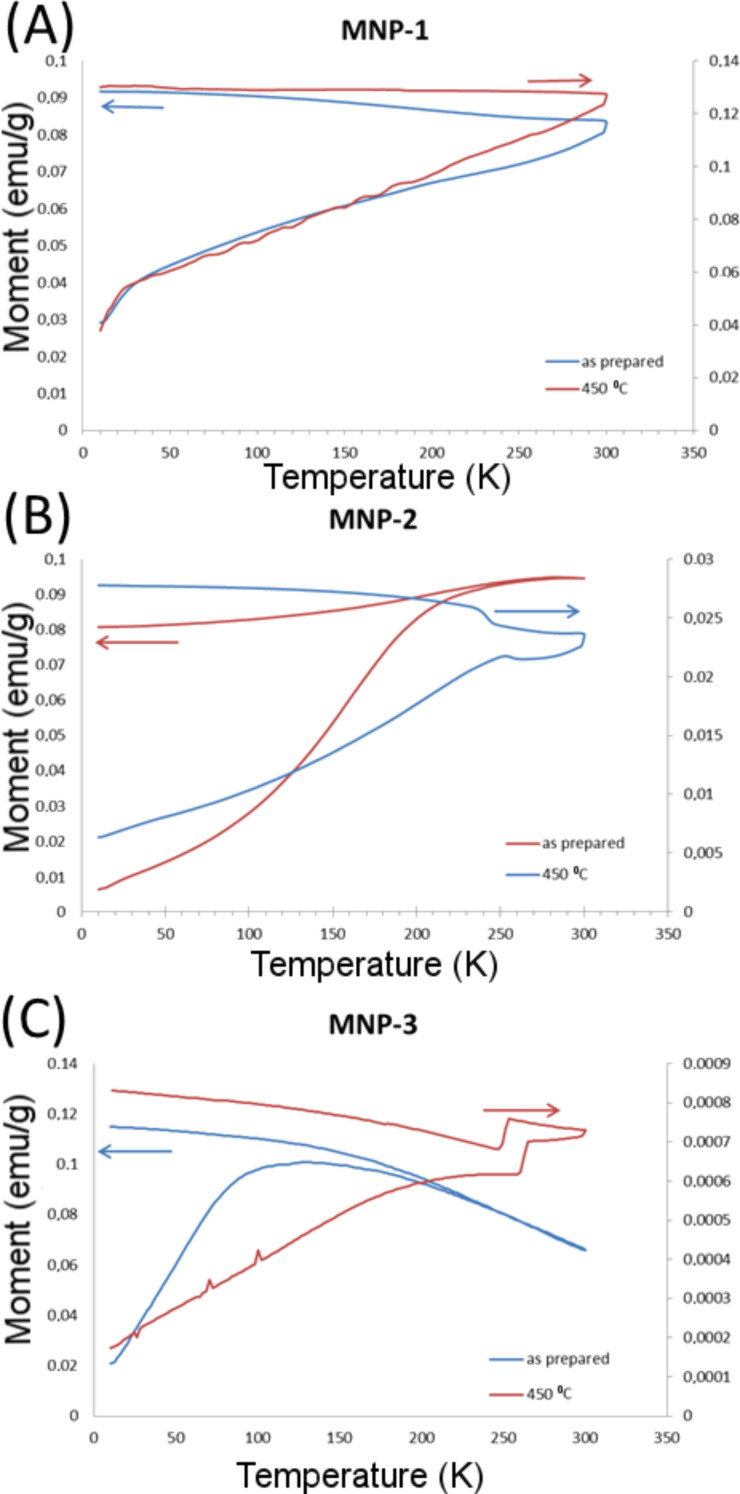
SQUID measurements for (A) MNP-1, (B) MNP-2, and (C) MNP-3.

The superconducting quantum interference device (SQUID) experiments were performed in a magnetic field of 50 Oe in the temperature range 10–300 K using zero-field cooled (ZFC) and field cooled (FC) measurement protocols. In the ZFC protocol, the sample is cooled from room temperature to 10 K in zero field. At low temperature the magnetic field is applied and the magnetization is recorded with increasing temperature. After reaching 300 K, the sample is re-cooled in the same field is applied while recording the FC magnetization (see Figure 8).

The magnetic behavior of the pure Fe_3_O_4_ sample appears largely unaffected by annealing in the measured temperature range (10–300 K), except for a clear reduction of the magnitude of the magnetic response (*M*/*H*) by a factor of about 0.6.

The Fe_3_O_4_/Fe-ox sample is quite strongly affected by the annealing and the particles are to some extent transformed to hematite by this process. This was learned from the appearance of anomalies in the *M* vs *T* curves near 250 K, reflecting the Morin transition [40]. Also, the magnetic moment of the annealed sample is significantly lower and the blocking temperature can be estimated to appear at a higher temperature than in the reference sample. The magnetic response (*M*/*H*) is decreased by a factor of about 0.2 in the annealed compared to the reference sample.

After annealing at 450 °C, the Fe_3_O_4_/Fe-ox/Ag sample has, to a large extent, transformed to hematite. This is seen from the appearance of a clear signature at about 250 K in the *M* vs *T* curves of the Morin transition of hematite. The original Fe_3_O_4_/Fe-ox/Ag sample shows a rather broad blocking temperature around 120 K. The magnetic response is decreased by a factor of about 0.07 between the annealed and the reference sample.

### Mössbauer spectroscopy

**Figure 9:**
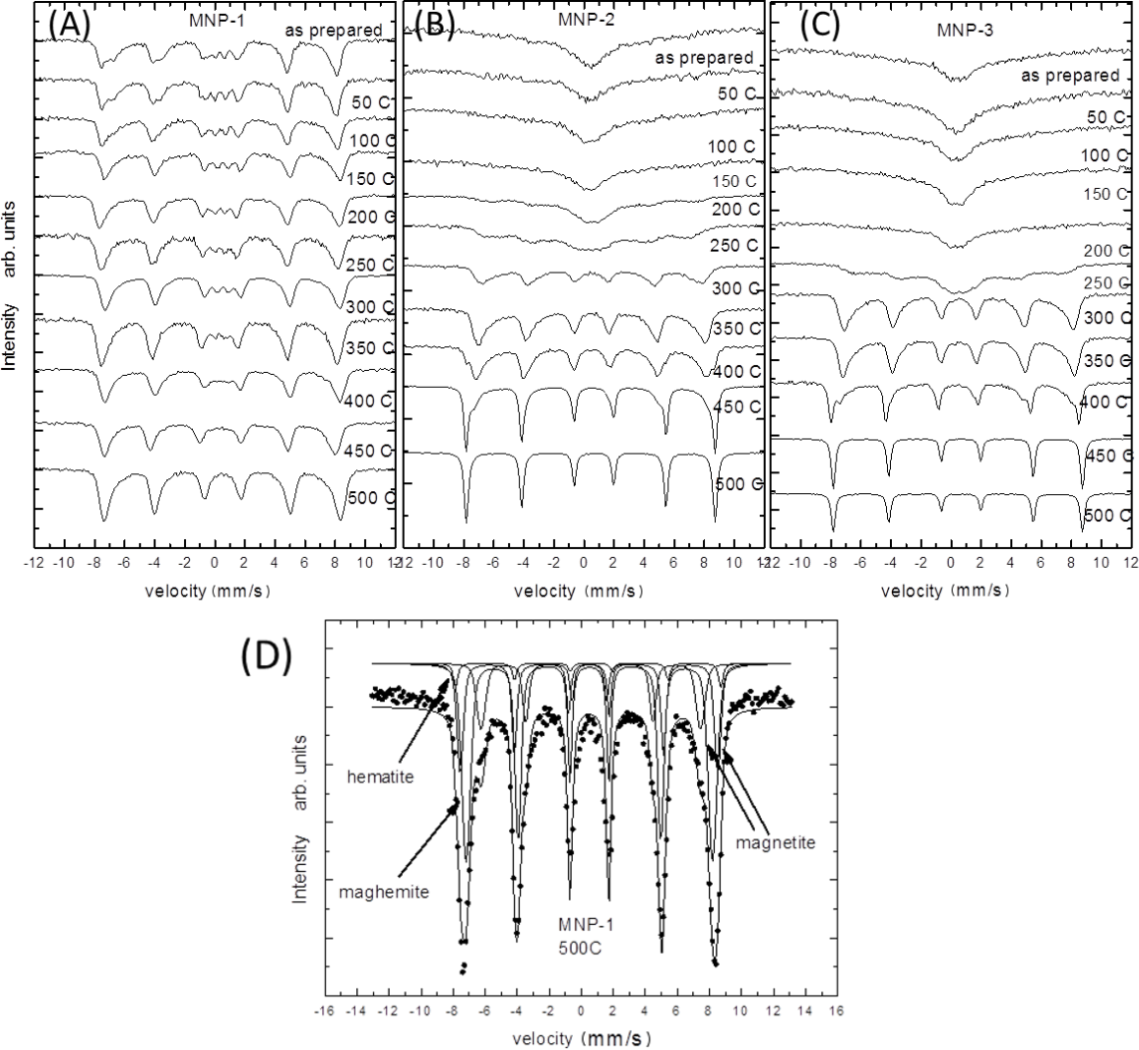
Mössbauer spectra of nanoparticles tested under different temperatures: (A) MNP-1, (B) MNP-2, (C) MNP-3 along with (D) examples of respective fits.

Mössbauer spectra were obtained with a standard spectrometer working in constant acceleration mode at RT. The results are plotted in series and depicted in Figure 9.

In Figure 9A, the spectra obtained from MNP-1 particles are collected. There, a slow transformation from the spectra typical for the bulk magnetite (as prepared), where two subspectra typical for Fe(A) and Fe(B) are present [41], is observed towards that characteristic for hematite or/and maghemite, where mostly one subspectrum with nearly average hyperfine field at RT is observed (particles heated at 500 °C) [42]. The measured RT Mössbauer spectra show that the particles are not in a superparamagnetic state, which means that all particles fall in size below superparamagnetic blocking temperature (*T*_B_) but the reminiscence of superparamagnetic doublet is detected in the center of the spectra [43]. For the particles heated at the highest temperature no doublet is seen, which can be the effect of the thermal phase transformation between Fe oxides. The spectra consist of a sextet with a relatively wide line at half maximum, which is connected with the overlapping of the magnetite, maghemite and hematite subspectra. The quantitative analysis suggests that the relative intensity ratio of Fe in these two phases is roughly equal to 4:78:18, respectively, at 500 °C (see Figure 9D).

In Figure 9B, the RT spectra of MNP-2 nanoparticles are depicted. There, the spectra transform much more significantly in comparison to the other series. The spectra of the as-prepared and low temperature heated particles are in the singlet form and only one broad line is seen. These types of magnetite particles are above the superaparamagnetic blocking temperature. With an increase in temperature during the heat treatment, the observed MS spectra become broader and finally, above 150 °C, they start to split into the sextet typical for the mixture of magnetite/maghemite [43]. After further temperature treatment above 400 °C, the hematite sharp lines start to appear as well. At 450 °C the coexistence of both phases is clearly seen. Finally, the simple sextet with very sharp lines is observed at 500 °C (with hyperfine parameters typical for hematite magnetic hyperfine field ≈52 T, quadruple splitting ≈0.2 mm/s, which is in good agreement with the bulk values (Figure 9D) [44–48].

Figure 9C presents the spectra of MNP-3 nanoparticles. The results show that the Ag shell does not significantly influence the thermal core stability because the oxidation process is as effective as it was observed for the uncoated particles. Therefore, the Ag shell extends the properties of magnetite nanoparticles, but the thermal stability of the core is not significantly protected. This suggests that the amount of oxide that is trapped in the particle volume is large enough for thermal oxidation maintenance, but no additional oxide can access the core.

It can be seen from the qualitative analysis that the MNP-2 particles are much more sensitive to the oxidation process in comparison to other particles. A possible explanation of the observed scenario is that the nanoparticles have different inherent structures that significantly influence the superparamagnetic blocking temperature. One argument is that the MNP-1 particles, which can be treated as single crystals, have been obtained. This can be seen in HRTEM studies [49]. Therefore, oxide penetration is hampered and becomes much slower. The stepwise decomposition of the Fe(acac)_3_ complex causes the presence of grain-like growth of each subsequent layer. This introduces many more grain boundaries and dislocations, and therefore, more room for oxide penetration, which significantly facilitates the oxidation process.

## Conclusion

The performed experiments show that thermal stability of magnetite nanoparticles is dependent on the fabrication procedure. The temperature scans, combined with structural and magnetic measurements, depict that nanoparticles of the same size but different oxidation state are not in the same magnetic state at RT. This is extremely important with regards to their application. A slow heat treatment allows modification of the oxidation state, but not of the particle size. The variations between magnetite, maghemite and hematite can lead to the fabrication of particles with desired and well-defined magnetic properties. It is shown that the magnetic state can be tuned after the fabrication process by post-treatment, which is in a good agreement previous studies. The lack of temperature treatment stability and the evolution of different oxides show the complexity behind the science regarding the nanostructure study. The development of various magnetic particles originates from the same compound, which makes the nanoscience more unpredictable but, at the same time, very interesting to study. Taking into account the different nature of the oxides present in the nanosized particles, further investigation of the nanoparticles in an external magnetic field is planned.
